# Exploratory Analysis of the Association Between Plasma Ceramide Alterations and Cognitive Dysfunction in Parkinson's Disease

**DOI:** 10.1111/cns.70082

**Published:** 2024-10-20

**Authors:** Xu Liu, Xuanjing Liu, Yuning Liu, Bo Yang, Yangdanyu Li, Fujia Li, Kun Qian, Xuesong Liu, Lishun Xiao, Guiyun Cui, Chuanying Xu

**Affiliations:** ^1^ Department of Neurology The Affiliated Hospital of Xuzhou Medical University Xuzhou Jiangsu China; ^2^ Department of Neurology The Second Affiliated Hospital of Xuzhou Medical University Xuzhou Jiangsu China; ^3^ Department of Neurology, the First Clinical College Xuzhou Medical University Xuzhou Jiangsu China; ^4^ Department of Biostatistics, School of Public Health Xuzhou Medical University Xuzhou Jiangsu China

**Keywords:** biomarker, ceramide, cognitive dysfunction, Parkinson's disease

## Abstract

**Objective:**

Prior research has underscored the importance of sphingolipid metabolism in Parkinson's disease (PD) pathogenesis. Our objective was to explore the associations between plasma ceramide levels and PD patients with cognitive dysfunction (PD‐CD).

**Methods:**

We enrolled two study populations from Eastern China and the Parkinson's Progression Markers Initiative (PPMI), comprising 290 (100 HCs, 160 PDs, and 30 MSAs) and 429 (125 HCs and 304 PDs) participants, respectively. The plasma levels of ceramides (Cer 16:0, Cer 18:0, Cer 24:0, and Cer 24:1) were tested via HPLC–MS/MS analysis.

**Results:**

Compared with those in the HC group, the plasma levels of Cer 18:0, Cer 24:1, Cer 16:0/Cer 24:0, Cer 18:0/Cer 24:0, and Cer 24:1/Cer 24:0 were higher in both the PD and MSA groups. Significant differences in the plasma levels of Cer 16:0/Cer 24:0, Cer 18:0/Cer 24:0, and Cer 24:1/Cer 24:0 were observed among the PD‐NC (PD with normal cognition), PD‐MCI (PD with mild cognitive impairment), and PDD (PD dementia) groups, with the PDD group exhibiting the highest levels. PD patients with higher baseline levels of plasma ceramides (specifically, Cer 18:0, Cer 16:0/Cer 24:0, Cer 18:0/Cer 24:0, and Cer 24:1/Cer 24:0) demonstrated accelerated cognitive decline compared with individuals who had lower baseline plasma ceramide levels during the 5‐year follow‐up period. A biomarker panel including Cer 18:0/Cer 24:0 and Cer 24:1/Cer 24:0 could effectively differentiate PD‐CD from PD‐NC with notable diagnostic accuracy.

**Conclusions:**

Our results indicate that plasma ceramide levels could potentially be used as diagnostic biomarkers for PD‐CD.

## Graphical abstract

PD patients with higher baseline plasma ceramide (Cer 18:0, Cer 16:0/Cer 24:0, Cer 18:0/Cer 24:0, and Cer 24:1/Cer 24:0) levels exhibited faster cognitive deterioration than those with lower baseline plasma ceramide levels over the 5‐year follow‐up.

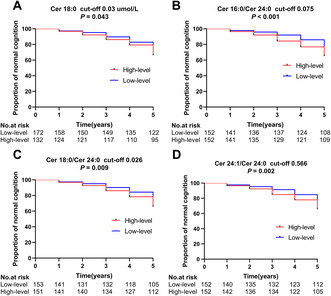

## 1 Introduction

Cognitive dysfunction (CD), which varies from mild cognitive impairment (MCI) to Parkinson's disease dementia (PDD), is one of the most prevalent and disabling symptoms in PD patients. CD may manifest at any stage of PD; approximately 25% of patients are diagnosed with MCI, and up to 83% eventually progress to dementia [1, 2]. The presence of CD markedly increases disability and overall functional decline, which tends to progress concomitantly with cognitive deterioration [3]. Recognizing Parkinson's disease with cognitive dysfunction (PD‐CD) in clinical practice currently relies mainly on relatively subjective neuropsychological evaluation, so there is a critical need for objective biomarkers that could be used to diagnose and predict cognitive impairment to enable early management for patients [4].

The pathophysiological mechanisms of CD in PD are not fully understood and may involve multiple mechanisms, including deposition of amyloid‐β (Aβ) in the cortex, abnormal aggregation of α‐synuclein (α‐syn), and dysregulated lipid metabolism [5]. Recent studies have provided insight into sphingolipids, a class of lipids, as potential prognostic biomarkers of PD [6, 7]. Ceramide is a central metabolite of sphingolipids and is involved in various cellular activities [8]. Elevated ceramide levels can promote Aβ production by stabilizing β‐secretase, while Aβ can further increase ceramide levels by activating sphingomyelinase, creating a positive feedback loop between ceramide and Aβ [9, 10]. Additionally, the disruption of ceramide homeostasis is associated with α‐synuclein aggregation [11, 12, 13] and enhances its toxicity [14]. Mingione et al. reported that the inhibition of ceramide synthesis mitigated α‐synuclein proteinopathy in a Parkinson's disease cellular model [15]. The evidence described above highlights the critical role of ceramides in the pathogenesis of PD‐CD. However, there is currently a lack of large‐sample longitudinal cohort studies exploring the relevant interplay between plasma ceramide levels and PD‐CD, so the relationship between the two is not yet clear.

In this exploratory analysis, we aimed to (1) tentatively compare plasma ceramide levels among healthy controls, idiopathic Parkinson's disease, and multiple system atrophy (atypical parkinsonism) groups and (2) explore whether plasma ceramide levels could be potential diagnostic and predictive biomarkers of cognitive impairment in PD patients in two study populations.

## 2 Methods

### 2.1 Study Participants in Eastern China

The study conducted in Eastern China was a cross‐sectional observational investigation that included 100 healthy controls (HCs) without significant organic diseases, 160 patients with PD, and 30 patients with multiple system atrophy (MSA) who visited the Department of Neurology, Affiliated Hospital of Xuzhou Medical University, from October 2021 to October 2023. MSA was diagnosed according to the established diagnostic guidelines [16, 17]. PD diagnosis followed the criteria set by the International Parkinson and Movement Disorder Society (MDS) in 2015 [18]. Patients with PD were subsequently categorized into three distinct groups, PD‐NC, PD‐MCI, and PDD, based on the diagnostic standards recommended by the MDS [4, 19]. The exclusion criteria for PD patients were as follows: (i) secondary or atypical parkinsonism; (ii) severe dysfunction of important organs such as the heart, lungs, brain, or kidneys; and (iii) dementia from causes other than PD, such as dementia with Lewy bodies. The study received ethical approval from the Ethics Committee of the Affiliated Hospital of Xuzhou Medical University (XYFY2023‐KL266‐01). This investigation adhered to the Declaration of Helsinki principles.

### 2.2 Study Participants in the PPMI Longitudinal Cohort Study

All clinical information from the Parkinson's Progression Markers Initiative (PPMI) was obtained after formal authorization (http://www.ppmi‐info.org) [20]. PD patients were initially enrolled in the PPMI cohort if they (a) were over 30 years of age, (b) were diagnosed with PD within 2 years without medication treatment, and (c) were at Hoehn and Yahr stages (H–Y stage) below 3. The exclusion criteria were as follows: (1) had a diagnosis of parkinsonism plus syndromes; (2) had a history of neurosurgical interventions; (3) had a history of psychiatric disorders, cancer, or severe cardiovascular conditions; and (4) were unable to complete gait or cognitive assessments. For this analysis, at baseline, 125 HCs and 304 PD patients with available clinical and laboratory data, including the levels of plasma ceramides (Cer 16:0, Cer 18:0, Cer 24:0, and Cer 24:1), were enrolled. Additionally, data from a 5‐year follow‐up period were included.

### 2.3 Clinical Data and Neurological Assessment

The demographic data recorded in this study included variables such as age, body mass index (BMI), sex, education level, levodopa‐equivalent daily dose (LEDD), and disease duration. When patients with PD were in the “Med‐Off” state, the MDS‐UPDRS III and H–Y stages were used to investigate the severity of motor symptoms and disease progression, respectively. Global cognitive function was evaluated by the Montreal Cognitive Assessment (MoCA) scale (“Med‐On” state). Sleep disorders were identified through the REM Sleep Behavior Disorder Screening Questionnaire (RBDSQ).

### 2.4 Plasma Ceramide Measurement

Venous blood samples were collected from all participating individuals in Eastern China during the early morning on an empty stomach. The plasma was then extracted via tubes filled with the anticoagulant dipotassium ethylenediaminetetraacetic acid (EDTA‐K2) supplied by CDHEALTH, China. Following extraction, these samples underwent a centrifugation process at 3000 rpm for 10 min at 4°C in a benchtop centrifuge (Thermo Fisher, United States) and were subsequently stored at −80°C. A 100 μL aliquot of the plasma sample was subsequently combined with 100 μL of an internal standard (IS) solution and 400 μL of a solution containing ethyl acetate and isopropanol (1:4, v/v). The mixture was vortexed for 5 min and then subjected to centrifugation at 11,000 rpm for 10 min at 4°C. Next, 300 μL of the obtained supernatant was subjected to further centrifugation under the same parameters, and 100 μL of this supernatant was set aside for analysis by mass spectrometry (MS).

HPLC–MS/MS analysis was conducted via a Waters TQD Triple Quadrupole mass spectrometer equipped with a positive‐mode electrospray ionization source. Chromatographic separation was achieved via an ACQUITY UPLC BEH C18 column (2.1 mm × 50 mm × 1.7 μm). Mobile phase A consisted of water infused with 1% formic acid and 5 mmol/L ammonium acetate, whereas mobile phase B included a mixture of isopropanol and acetonitrile (4:3, v/v) containing 5 mmol/L ammonium acetate. An injection volume of 15 μL was used alongside a flow rate of 0.3 mL/min. For ceramide quantification, the multiple reaction monitoring (MRM) technique was employed, which targets ceramides (Cer 16:0, Cer 18:0, Cer 24:0, and Cer 24:1).

The study protocols for the PPMI cohort are available online at: http://www.ppmi‐info.org.

### 2.5 Statistical Analysis

Continuous variables were tested for normality using the Shapiro–Wilk test; variables were analyzed with Student's *t* test and are presented as mean ± SD when normally distributed; alternatively, variables that were not normally distributed were evaluated by the Mann–Whitney *U* test and the Kruskal–Wallis test, and the results are expressed as median (interquartile range, IQR). Categorical data, presented as numbers (percentages), were compared via the *χ* ^2^ test. The correlation between plasma ceramide levels and cognitive status was determined via Spearman's rank correlation. In the PPMI cohort, patients with PD were classified into groups of high (> median value) and low (≤ median value) according to the median baseline plasma ceramide concentrations, and the proportion of individuals with normal cognition among these groups was assessed via the Kaplan–Meier method. All baseline variables with *p* values under 0.2 in the univariable logistic regression analysis were subsequently included in the multivariable logistic regression with backward elimination. The Cox model was used to identify potential independent risk factors for PD‐CD over a follow‐up period of 1–3 years. Receiver operating characteristic (ROC) curve analysis was performed to examine the diagnostic performance of biomarkers or combined markers in differentiating PD‐CD from PD‐NC. Statistical significance was set at a two‐sided *p* < 0.05. Statistical assessments were carried out with SPSS version 26.0 (SPSS, Inc.), OriginPro 2021 (OriginLab Corp.), and GraphPad Prism version 8.0 (GraphPad Software).

## 3 Results

### 3.1 Baseline Characteristics of all Subjects

**FIGURE 1:**
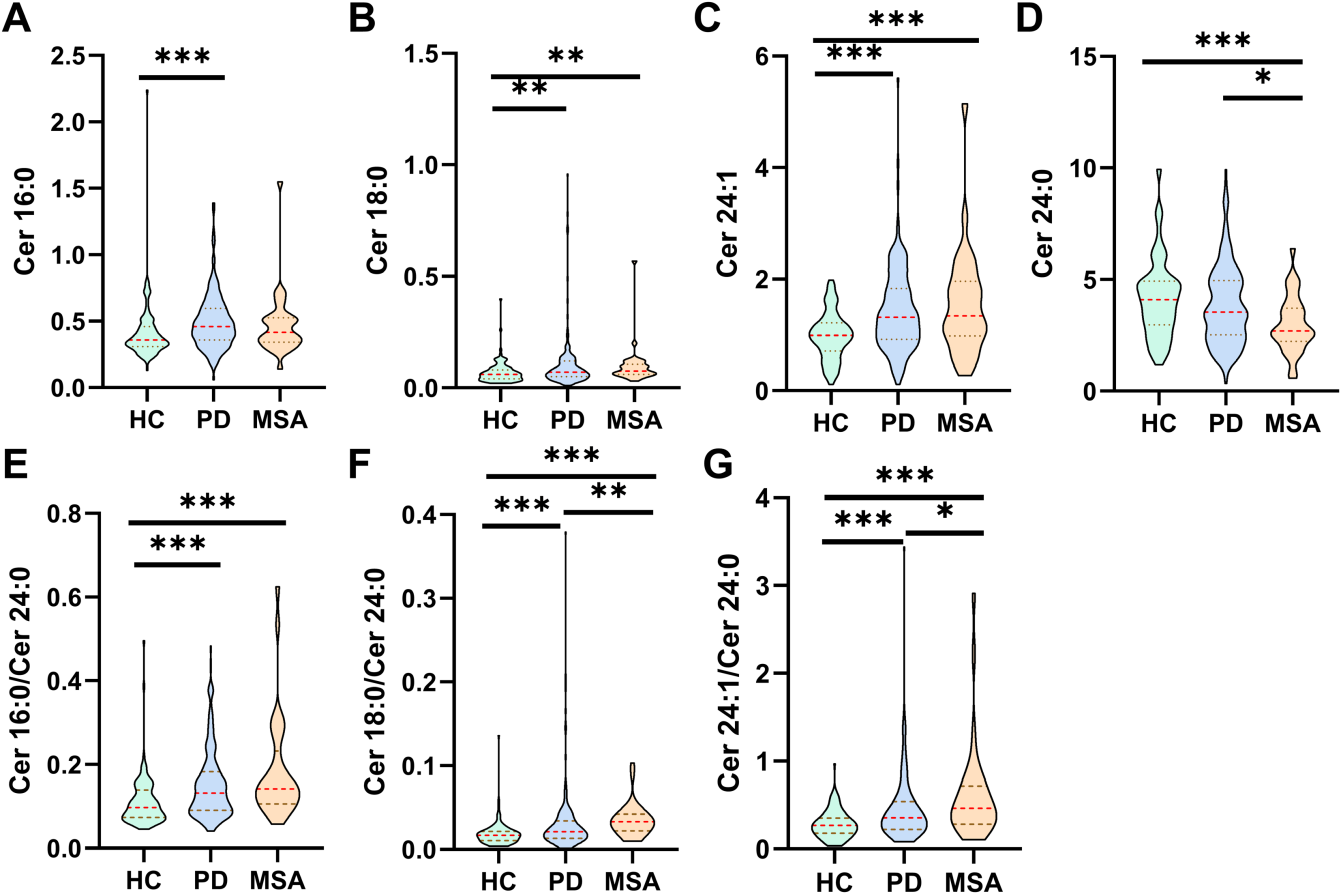
Comparisons of the levels of plasma ceramides among HC, PD, and MSA groups from Eastern China. *p < 0.05, **p < 0.01, ***p < 0.001.

Table 1 and Figure 1 Display the demographic and clinical data for the HC, PD, and MSA groups in Eastern China. No significant differences in sex, BMI, MoCA score, or LEDD were observed among these groups. However, the PD and MSA groups differed significantly in terms of age, disease duration, H–Y stage, and the MDS‐UPDRS III score. Additionally, these groups demonstrated plasma levels of Cer 18:0, Cer 24:1, Cer 16:0/Cer 24:0, Cer 18:0/Cer 24:0, and Cer 24:1/Cer 24:0 that were higher than those of the HC group, with the MSA group exhibiting the highest values of Cer 18:0/Cer 24:0 and Cer 24:1/Cer 24:0. In contrast, the plasma Cer 24:0 level was significantly lower in the MSA group than in the HC group. Furthermore, similar results revealed that the PD group in the PPMI cohort had levels of plasma Cer 16:0/Cer 24:0, Cer 18:0/Cer 24:0, and Cer 24:1/Cer 24:0 that were significantly higher than those of the HC group, as shown in Table S1.

**TABLE 1:** Demographic and clinical characteristics of all subjects from Eastern China.

Variables	HC (*N* = 100)	PD (*N* = 160)	MSA (*N* = 30)
Age (years)	63.96 ± 10.58	68 (9)^a^, *	63.05 ± 7.01^c^, *
Male sex, *n* (%)	42 (42%)	90 (52.94%)	16 (53.33%)
BMI, kg/m^2^	24.63 (4.06)	24.08 ± 3.40	24.67 ± 3.67
Education, years	9 (3)	9 (6)^a^, ***	7.97 ± 4.67
MoCA score	NA	19 (10)	16 (10)
Disease duration, years	NA	5 (5)	3 (3)^c^, *
LEDD (mg)	NA	600 (525)	550.37 ± 343.05
MDS‐UPDRS III score	NA	38 (33)	52.73 ± 22.32^c^, *
H–Y stage	NA	2.5 (1.0)	3 (1.5)^c^, ***
Cer 16:0, μmol/L	0.36 (0.15)	0.46 (0.24)^a^, ***	0.42 (0.18)
Cer 18:0, μmol/L	0.06 (0.04)	0.07 (0.07)^a^, **	0.08 (0.05)^b^, **
Cer 24:0, μmol/L	4.06 (1.96)	3.55 (2.44)	2.70 (1.49)^b^, ** ^,^ ^c^, *
Cer 24:1, μmol/L	1.00 (0.50)	1.32 (0.91)^a^, ***	1.34 (0.98)^b^, ***
Cer 16:0/Cer 24:0	0.10 (0.07)	0.13 (0.09)^a^, ***	0.14 (0.13)^b^, ***
Cer 18:0/Cer 24:0	0.02 (0.01)	0.02 (0.02)^a^, ***	0.03 (0.02)^b^, *** ^,^ ^c^, **
Cer 24:1/Cer 24:0	0.27 (0.18)	0.35 (0.32)^a^, ***	0.46 (0.43)^b^, *** ^,^ ^c^, *

Abbreviations: BMI, body mass index; Cer, ceramide; HC, healthy control; H–Y, Hoehn and Yahr; LEDD levodopa‐equivalent daily dose; MDS‐UPDRS III, movement disorder society unified parkinson's disease rating scale part III; MoCA, montreal cognitive assessment; MSA, multiple system atrophy; PD, parkinson's disease.

^a^ PD versus HC.

^b^ MSA versus HC.

^c^ MSA versus PD.

* *p* < 0.05,

** *p* < 0.01,

*** *p* < 0.001.

### 3.2 Comparisons of Plasma Ceramide Levels Based on Cognitive Status

**FIGURE 2:**
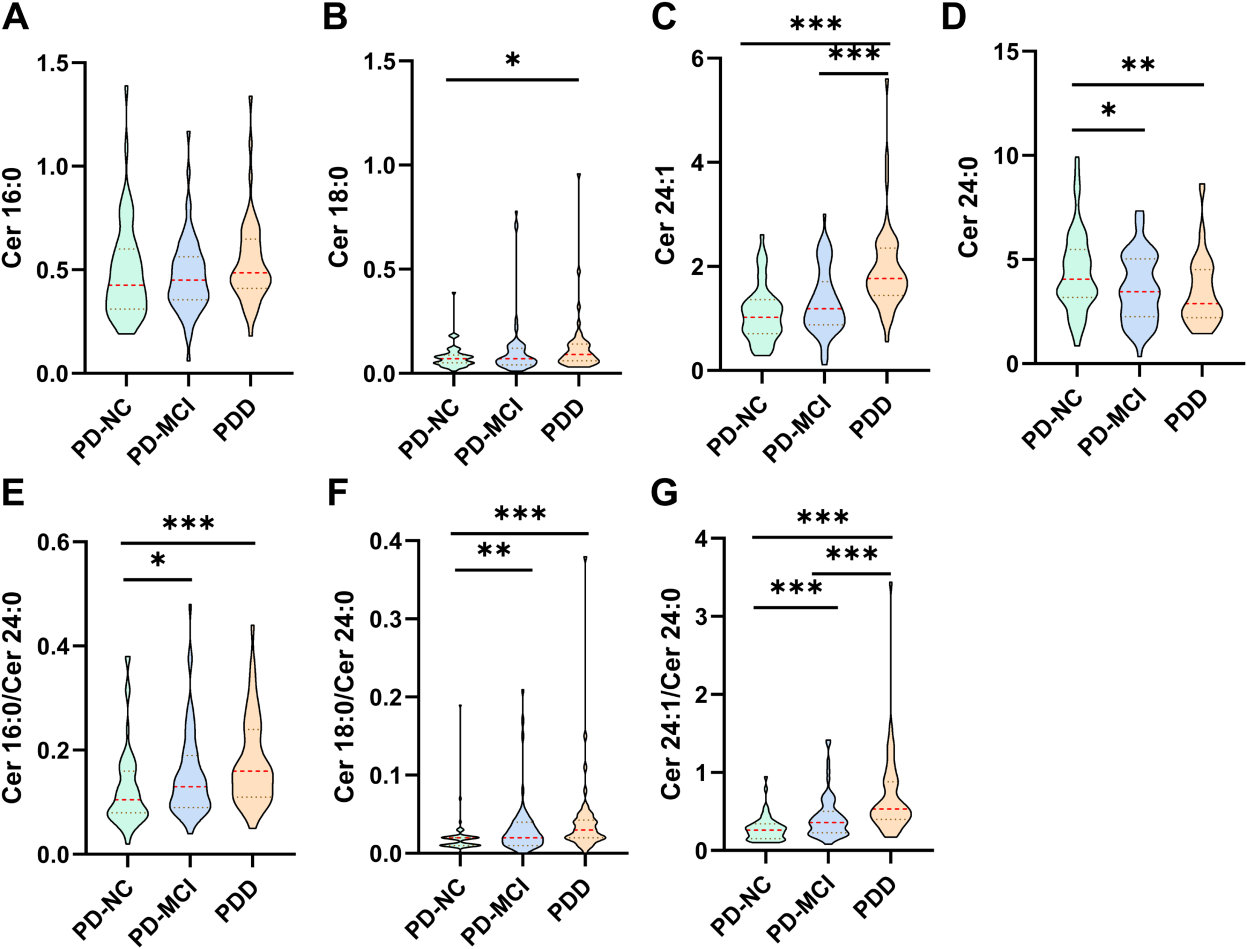
Comparisons of the levels of plasma ceramides among PD‐NC, PD‐MCI, and PDD groups from Eastern China. *p < 0.05, **p < 0.01, ***p < 0.001.

No substantial distinctions were detected between the PD‐NC and PD‐CD groups in the categories of sex, BMI, LEDD, RBDSQ scores, or Cer 16:0 and Cer 18:0 levels. However, the differences between the PD‐NC and PD‐CD groups were significant with respect to age, education years, MoCA score, MDS‐UPDRS III score, and the H–Y stage. The PD‐CD group also presented notably greater levels of Cer 24:1, Cer 16:0/Cer 24:0, Cer 18:0/Cer 24:0, and Cer 24:1/Cer 24:0 than did the PD‐NC group. More precisely, in terms of cognitive status, significant differences were found in the plasma levels of Cer 16:0/Cer 24:0, Cer 18:0/Cer 24:0, and Cer 24:1/Cer 24:0 across the PD‐NC, PD‐MCI, and PDD groups. Compared with the PD‐NC group, the PDD group presented significantly elevated levels of Cer 18:0 and Cer 24:1. Conversely, the highest plasma Cer 24:0 level was observed in the PD‐NC group (Table 2, Figure 2). In addition, patients in the PPMI cohort with MoCA scores ≤ 25 presented significantly higher levels of Cer 18:0/Cer 24:0 and Cer 24:1/Cer 24:0 than those with MoCA scores > 25 [21, 22, 23], as detailed in Table S2.

**TABLE 2:** Demographic and clinical characteristics of PD patients from eastern china grouping by cognitive status.

Variables	PD‐NC (*N* = 52)	PD‐CD (*N* = 108)	PD‐MCI (*N* = 62)	PDD (*N* = 46)
Age (years)	64.12 ± 8.08	69 (8)^a^, ***	67.00 ± 8.09^b^, *	70.20 ± 5.28^c^, *** ^,^ ^d^, *
Male sex, *n* (%)	34 (65.38%)	56 (51.85%)	40 (66.67%)	16 (34.78%)^c^, ** ^,^ ^d^, **
BMI, kg/m^2^	24.28 ± 3.06	23.90 ± 3.55	24.20 ± 3.51	23.49 ± 3.60
Education, years	9 (3)	6 (9)^a^, ***	8 (6)^b^, ***	5 (8.25)^c^, *** ^,^ ^d^, ***
MoCA score	23.31 ± 3.36	16 (7)^a^, ***	19.5 (6.0)^b^, ***	13 (7)^c^, *** ^,^ ^d^, ***
Disease duration, years	5 (5)	5 (5)	4 (6)	7.00 (5.25)^c^, * ^,^ ^d^, *
LEDD (mg)	540.87 ± 327.92	636.14 ± 357.51	579.78 ± 373.48	712.11 ± 323.36^c^, * ^,^ ^d^, *
MDS‐UPDRS III score	28 (20)	44.0 (36.5)^a^, ***	35.00 (31.25)^b^, *	55 (28)^c^, *** ^,^ ^d^, ***
RBDSQ score	3 (5)	2 (5)	2 (5)	5.5 (5.0)^c^, * ^,^ ^d^, ***
H‐Y stage	2 (1)	2.5 (1.0)^a^, **	2.5 (1.0)	3 (0.75)^c^, *** ^,^ ^d^, ***
Cer 16:0, μmol/L	0.43 (0.29)	0.47 (0.22)	0.45 (0.21)	0.49 (0.24)
Cer 18:0, μmol/L	0.07 (0.03)	0.08 (0.08)	0.07 (0.08)	0.09 (0.08)^c^, *
Cer 24:0, μmol/L	4.21 (2.32)	3.37 (2.55)^a^, **	3.46 (2.78)^b^, *	2.88 (2.31)^c^, **
Cer 24:1, μmol/L	1.02 (0.68)	1.46 (1.00)^a^, ***	1.19 (0.83)	1.77 (0.91)^c^, *** ^,^ ^d^, ***
Cer 16:0/Cer 24:0	0.11 (0.09)	0.14 (0.10)^a^, ***	0.13 (0.09)^b^, *	0.16 (0.13)^c^, ***
Cer 18:0/Cer 24:0	0.02 (0.01)	0.03 (0.03)^a^, ***	0.02 (0.02)^b^, **	0.03 (0.03)^c^, ***
Cer 24:1/Cer 24:0	0.25 (0.19)	0.43 (0.35)^a^, ***	0.36 (0.28)^b^, ***	0.53 (0.48)^c^, *** ^,^ ^d^, ***

Abbreviations: PD‐NC, PD with normal cognition; PD‐MCI, PD with mild cognitive impairment; PDD, PD with dementia; PD‐CD, PD with cognitive dysfunction (including PD‐MCI and PDD); RBDSQ REM, Sleep Behavior Disorder Screening Questionnaire.

^a^ PD‐CD versus HC.

^b^ PD‐MCI versus HC.

^c^ PDD versus HC.

^d^ PDD versus PD‐MCI.

* *p* < 0.05,

** *p* < 0.01,

*** *p* < 0.001.

### 3.3 Correlations Between Plasma Ceramide Levels and Cognitive Dysfunction

**FIGURE 3:**
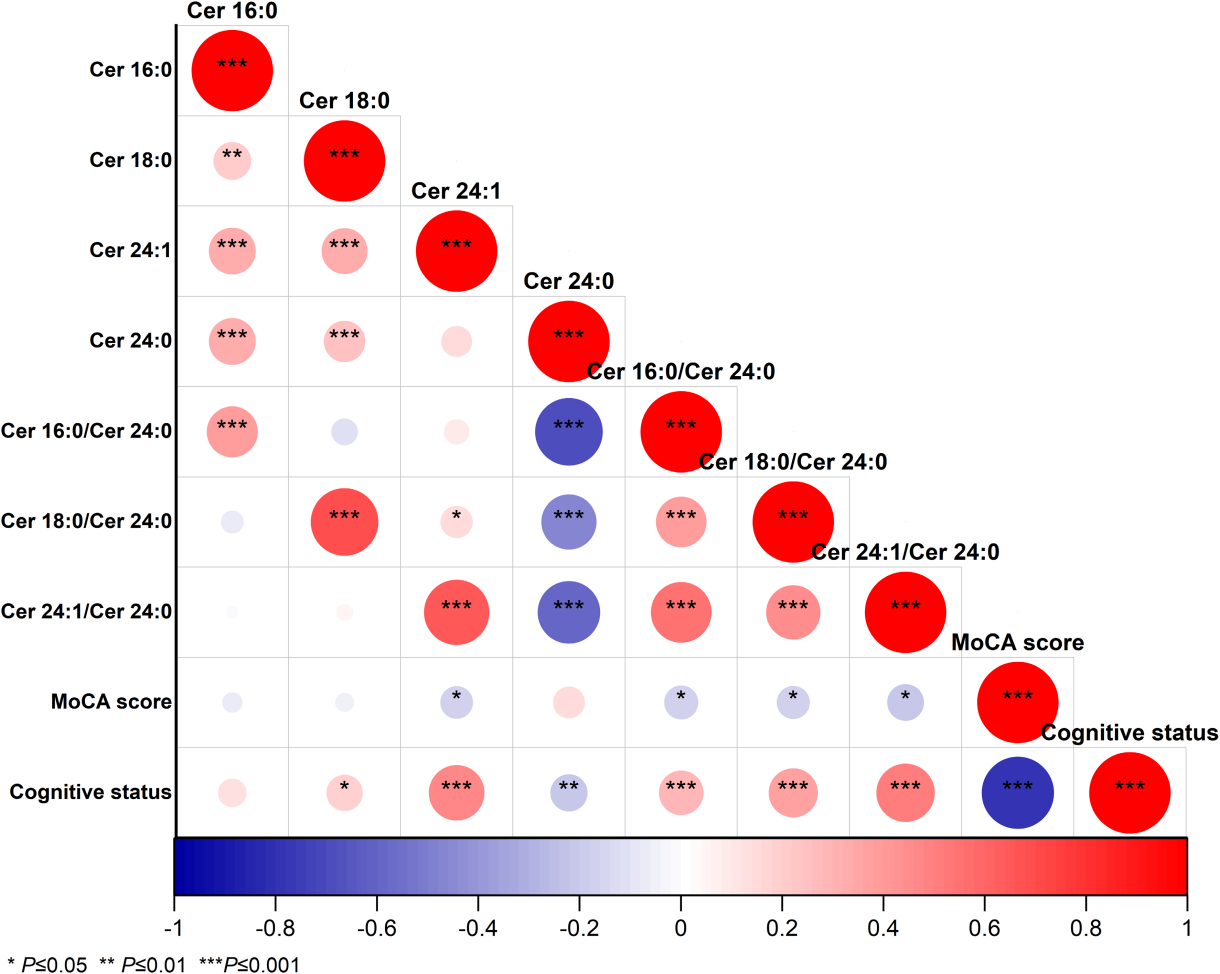
Correlations among plasma ceramides, cognitive status, and MoCA score in PD patients from Eastern China. The circle's size represents the value of the correlation coefficient determined through Spearman's correlation analysis. Positive correlations are indicated in red, while negative correlations are depicted in blue.

As illustrated in Figure 3, among the four plasma biomarkers (Cer 16:0, Cer 18:0, Cer 24:0, and Cer 24:1), only Cer 24:0 and Cer 24:1 showed no significant correlation; all others were significantly and positively correlated. Furthermore, the plasma levels of Cer 24:1, Cer 16:0/Cer 24:0, Cer 18:0/Cer 24:0, and Cer 24:1/Cer 24:0 were significantly negatively correlated with the MoCA score and positively correlated with cognitive dysfunction. Notably, the plasma level of Cer 24:0 was significantly inversely correlated with cognitive dysfunction (*r* = −0.21, *p* = 0.008).

**FIGURE 4:**
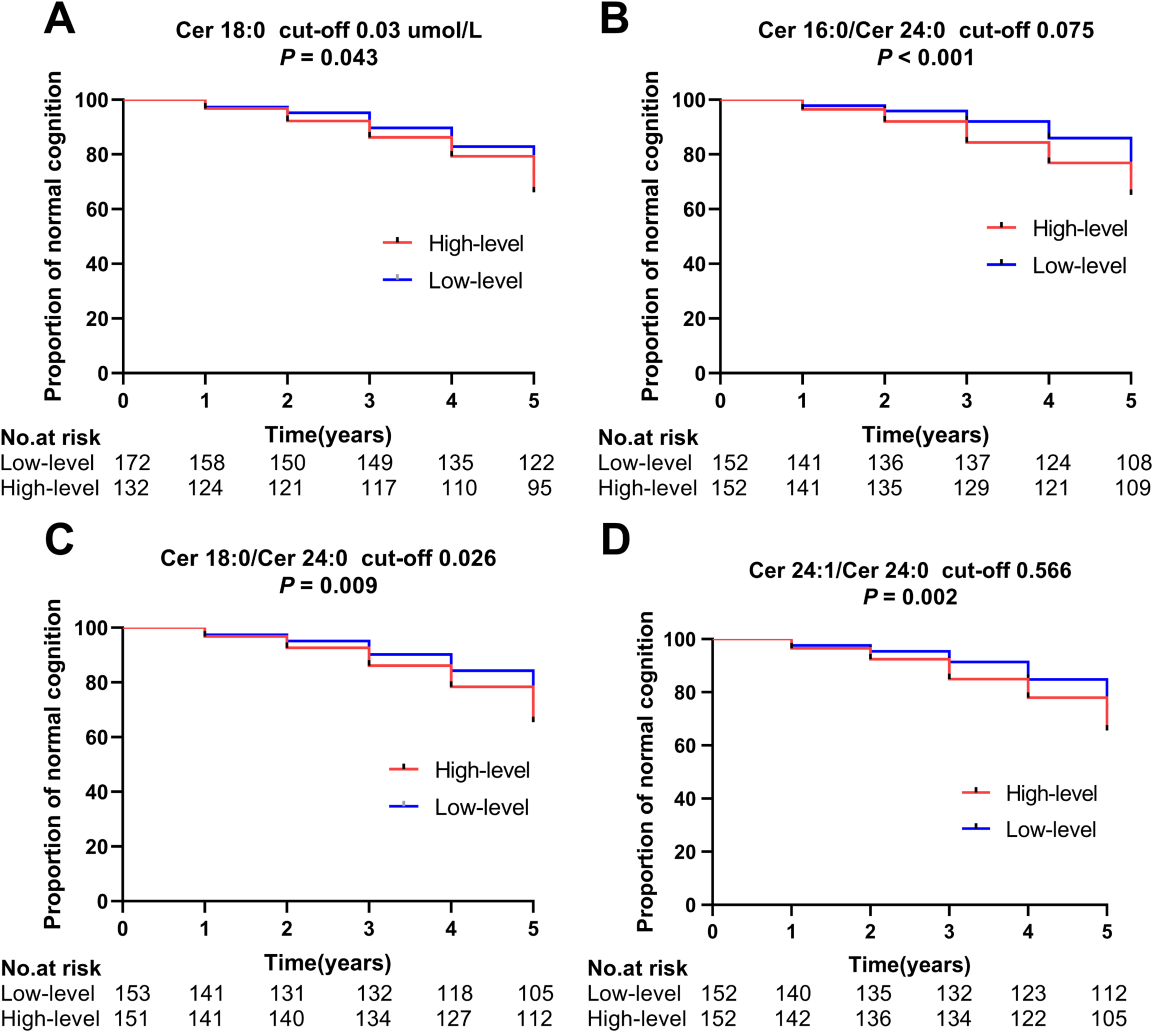
Associations between the proportion of normal cognition and plasma ceramide grouping by baseline median concentrations in the Kaplan – Meier method in the PPMI cohort.

In the PPMI cohort, Kaplan–Meier estimates over the 5‐year follow‐up revealed that the plasma levels of Cer 18:0, Cer 16:0/Cer 24:0, Cer 18:0/Cer 24:0, and Cer 24:1/Cer 24:0 in the high‐level (> baseline median) group presented a significantly lower proportion of individuals with normal cognition than did those in the low‐level (≤ baseline median) group (Figure 4, Figure S1).

### 3.4 Risk Factors for Cognitive Dysfunction in PD Patients

Univariable logistic regression analyses revealed that age, education level, MDS‐UPDRS III score, H–Y stage, MoCA score, and plasma levels of Cer 24:0, Cer 24:1, Cer 16:0/Cer 24:0, Cer 18:0/Cer 24:0, and Cer 24:1/Cer 24:0 were correlated with cognitive dysfunction in PD patients from Eastern China. Subsequent multivariate logistic regression analysis revealed that the plasma levels of Cer 24:0, Cer 24:1, and Cer 24:1/Cer 24:0 remained significantly correlated with PD‐CD, suggesting that these biomarkers are potential independent risk factors for PD‐CD (Table 3). Cox regression analyses confirmed that age, LEDD score, MDS‐UPDRS III score, H–Y stage, and plasma levels of Cer 16:0/Cer 24:0 and Cer 18:0/Cer 24:0 were significantly related to PD‐CD in the PPMI cohort **(**Table S3).

**TABLE 3:** Logistic regression to identify factors for cognitive dysfunction in PD patients from Eastern China.

Variables	Univariable analysis		Multivariable analysis^a^		Multivariable analysis^b^	
	*B*	*p*	*B*	*p*	*B*	*p*
Age, years	0.072	**0.002**	—		—	
Sex	−0.562	0.108	—		—	
BMI, kg/m^2^	−0.056	0.257	—		—	
LEDD (mg)	0.001	0.103	—		—	
Disease duration, years	−0.021	0.475	—		—	
Cer 16:0, μmol/L	0.202	0.804	—		—	
Cer 18:0, μmol/L	4.817	0.087	—			
Cer 24:0, μmol/L	−0.264	**0.007**	−0.399	**0.001**		
Cer 24:1, μmol/L	1.244	**< 0.001**	1.643	**< 0.001**		
Cer 16:0/Cer 24:0	5.943	**0.018**			—	
Cer 18:0/Cer 24:0	27.725	**0.021**			—	
Cer 24:1/Cer 24:0	5.007	**< 0.001**			4.885	**< 0.001**

*Note:* Due to severe collinearity issues among Cer 16:0, Cer 18:0, Cer 24:0, Cer 24:1 and their ratios Cer 16:0/Cer 24:0, Cer 18:0/Cer 24:0, Cer 24:1/Cer 24:0 (the tested variance inflation factor exceeding 10), they are not suitable for simultaneous inclusion in the multivariable regression model. Significance of bold values represents the *p* value < 0.05.

^a^ Including factors for multivariable analysis: Age, sex, LEDD, Cer 18:0, Cer 24:0, Cer 24:1.

^b^ Including factors for multivariable analysis: Age, sex, LEDD, Cer 16:0/Cer 24:0, Cer 18:0/Cer 24:0, Cer 24:1/Cer 24:0.

### 3.5 Diagnostic Accuracy

**FIGURE 5:**
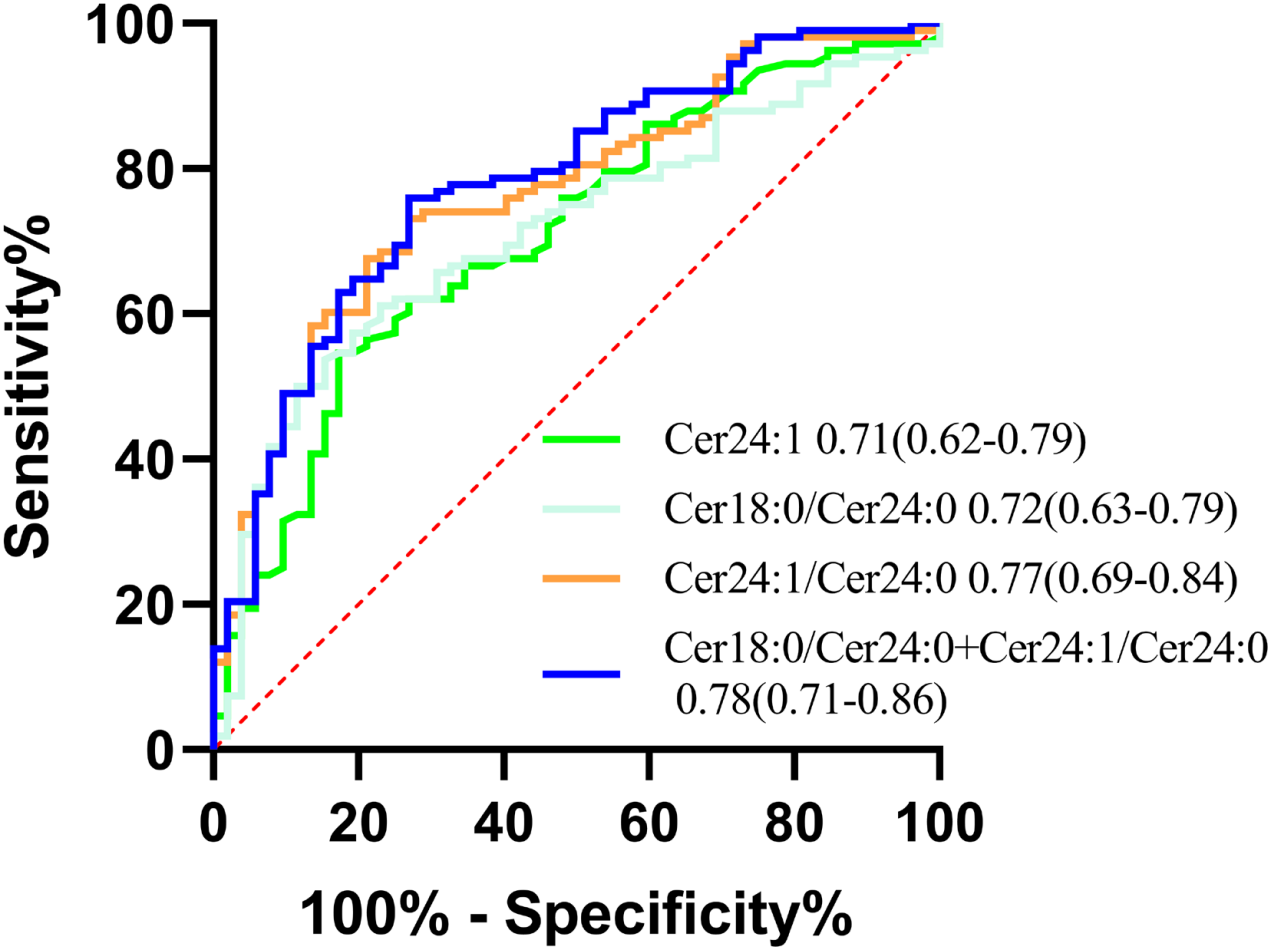
ROC curves of plasma ceramides and MoCA score in differentiating PD‐CD from PD‐NC in participants from Eastern China.

Plasma levels of Cer 24:1, Cer 18:0/Cer 24:0, and Cer 24:1/Cer 24:0 effectively differentiated PD‐CD patients from PD‐NC patients, demonstrating their relative diagnostic reliability (AUC 0.71, 95% CI 0.62–0.79; AUC xx 95% CI; AUC 95% CI, respectively). By enhancing the differentiation between PD‐CD and PD‐NC, the combined analysis of plasma Cer 18:0/Cer 24:0 and Cer 24:1/Cer 24:0 levels yielded high diagnostic accuracy (AUC, 95% CI) (Figure 5).

## 4 Discussion

Recent studies have proposed broadening our comprehension of Parkinson's disease beyond mere proteinopathy to include aspects of lipidopathy; this shift highlights the need for increased focus on the role of sphingolipid metabolism in PD [7, 24, 25]. Ceramide, a pivotal molecule within the sphingomyelin signaling pathway, acts as a second messenger and is crucial for cellular homeostasis [8, 26]. In the present study, we conducted an exploratory analysis to compare plasma ceramide levels among HC, PD, and MSA groups and first investigated the diagnostic and predictive roles of ceramide in PD patients with cognitive dysfunction in a longitudinal cohort study. Our findings revealed that the plasma levels of Cer 18:0, Cer 24:1, Cer 16:0/Cer 24:0, Cer 18:0/Cer 24:0, and Cer 24:1/Cer 24:0 in the PD and MSA groups were significantly greater than those in the HC group. A significant positive association was established between these plasma biomarkers and PD‐CD. Higher baseline plasma ceramide levels were associated with greater cognitive impairment in PD patients. Furthermore, these biomarkers demonstrated reliable diagnostic accuracy in differentiating PD‐CD from PD‐NC.

Prior studies have established a significant relationship between ceramides and Alzheimer's disease (AD) [9, 27, 28, 29], and Mielke et al. demonstrated that plasma ceramide levels can be used to predict hippocampal volume loss [30]. Motivated by these findings, we conducted an exploratory study to assess the role of plasma ceramide levels in PD patients with cognitive dysfunction. Our results indicated that the PD‐CD group presented significantly higher levels of plasma Cer 24:1, Cer 16:0/Cer 24:0, Cer 18:0/Cer 24:0, and Cer 24:1/Cer 24:0 than did the PD‐NC group in Eastern China, which aligns with the findings of a previous small sample size cross‐sectional study [31]. Interestingly, the plasma Cer 24:0 level was notably greater in the HC group than in the MSA group and in the PD‐NC group than in the PD‐CD group. Furthermore, the plasma level of Cer 24:0 was negatively correlated with cognitive dysfunction; this is in contrast with the positive correlation observed with other biomarkers, suggesting an opposing role for Cer 24:0 in PD development. This hypothesis is supported by evidence showing distinct biophysical effects of different ceramide lengths and species [32, 33]. In addition, since the PPMI cohort consists entirely of de novo PD patients, the proportion of patients with cognitive impairment at baseline is very low; as an alternative, we chose to compare the levels of plasma ceramides on the basis of MoCA scores at the third‐year follow‐up time point. The differences in the results between the two included study populations may be explained by differences in race, the fact that PD patients in the PPMI cohort were in early H–Y stages and contained a lower proportion of individuals with cognitive impairment.

Notably, Kaplan–Meier estimates demonstrated that the high‐level group of plasma Cer 16:0/Cer 24:0, Cer 18:0/Cer 24:0, and Cer 24:1/Cer 24:0 ratios, rather than the Cer 16:0, Cer 24:0, and Cer 24:1 ratios, had faster cognitive decline. These results indicate that we need to pay more attention to the values of ratios and the interplay of biomarkers when conducting biomarker research. This finding is similar to the consensus that the cerebral spinal fluid (CSF) Aβ42/Aβ40 ratio has greater diagnostic accuracy for AD than the CSF Aβ42 or Aβ40 values alone.

The multivariate logistic regression results from this study indicate that the plasma levels of Cer 18:0, Cer 24:1, Cer 24:0, Cer 18:0/Cer 24:0, and Cer 24:1/Cer 24:0 are independent risk factors for PD‐CD. Specifically, higher levels of plasma Cer 18:0, Cer 24:1, Cer 18:0/Cer 24:0, and Cer 24:1/Cer 24:0 are associated with an increased risk of cognitive impairment in PD patients. Conversely, higher levels of plasma Cer 24:0 are associated with a reduced risk of cognitive impairment in PD patients. These findings suggest that, clinically, greater attention should be given to the detrimental effects of high plasma levels of Cer 18:0, Cer 24:1, Cer 24:0, Cer 18:0/Cer 24:0, and Cer 24:1/Cer 24:0 on cognitive functions in PD. Currently, the MoCA scale is commonly used in the clinical assessment of cognitive impairment in PD patients. Given the subjective bias of the MoCA scale and its sensitivity to education levels, objective biomarkers that can identify cognitive decline are urgently needed. Our findings indicate that the plasma levels of Cer 24:1, Cer 18:0/Cer 24:0, and Cer 24:1/Cer 24:0 may serve as diagnostic biomarkers for PD‐CD with relatively strong diagnostic reliability. Taking the plasma levels of Cer 18:0/Cer 24:0 and Cer 24:1/Cer 24:0 together enhances diagnostic precision, demonstrating the superior ability of the biomarker panel to distinguish between PD‐CD and PD‐NC compared with single biomarkers.

Previous studies have indicated that ceramides might contribute to cognitive impairment in PD patients through several mechanisms. Notably, approximately 50% of patients with PDD exhibit significant accumulation of Aβ plaques [34]. Elevated ceramide levels increase Aβ production, whereas oligomerization of Aβ further increases ceramide levels by activating sphingomyelinases, enzymes that catalyze the breakdown of sphingomyelin into ceramide [10]. Furthermore, past research has demonstrated a strong correlation between the progression of fibrillar α‐synuclein pathology from the brainstem to the limbic and neocortical regions and the onset of dementia in PD patients [34]. Mutations in the GBA gene, which is crucial for ceramide metabolism, result in more severe cognitive dysfunction in PD patients [35, 36], likely due to reduced glucocerebrosidase (GCase) activity, which disrupts the ceramide balance and facilitates α‐synuclein accumulation in neurons [37, 38, 39]. This evidence underscores the importance of genetic factors involved in sphingolipid metabolism in the mechanism of PD‐CD. In addition, emerging research links plasma ceramides with systemic inflammation and insulin resistance, both of which are closely associated with cognitive impairment in PD patients [40, 41, 42].

The interpretation of our findings warrants caution because of several limitations in our study. Initially, we conducted a preliminary comparison of plasma ceramide levels between the PD and MSA groups. However, given the small sample size, extensive studies with larger samples and longitudinal designs are imperative to ascertain the distinct role of plasma ceramides in PD and atypical parkinsonism more accurately. Furthermore, subsequent research on the role of plasma ceramide levels in PD patients with cognitive deficits should account for the influence of the GBA and APOE genotypes. Finally, the inclusion of other biomarkers related to cognitive impairment, such as fluid α‐synuclein, Aβ42/Aβ40, neurofilament light (NfL), and p‐tau 217, followed by the identification of the most effective individual biomarkers or a combination thereof, will have great clinical significance in the diagnosis and prediction of cognitive impairment in PD patients.

In conclusion, our study revealed that the plasma levels of Cer 16:0/Cer 24:0, Cer 18:0/Cer 24:0, and Cer 24:1/Cer 24:0 are significantly elevated in patients with PD and MSA. Furthermore, elevated baseline plasma ceramide levels are correlated with accelerated cognitive decline in PD patients. Consequently, these plasma ceramides could serve as promising, noninvasive diagnostic and prognostic biomarkers of PD‐associated cognitive impairment. Expanding our understanding of the role of ceramide metabolism could significantly increase our understanding of its pathogenesis and inform therapeutic strategies for cognitive dysfunction in PD patients.

## Author Contributions

Research project: X.L., X.j.L., Y.L., G.C., C.X. Data acquisition: X.L., X.j.L., Y.L., B.Y., Y.L., F.L., K.Q. Statistical analysis: X.L., X.j.L., X.s.L., L.X. Writing – original draft preparation: X.L. Writing – review and editing: G.C., C.X. All authors have approved the final version of the work.

## Ethics Statement

The study received ethical approval from the Ethics Committee of the Affiliated Hospital of Xuzhou Medical University (XYFY2023‐KL266‐01). This investigation adhered to Declaration of Helsinki principles.

## Conflicts of Interest

The authors declare no conflicts of interest.

## Supporting information


Appendix S1.

## Data Availability

Data used in the present study from the PPMI cohort are available online (https://www.ppmi‐info.org/accessdata‐specimens/download‐data/), and data from Eastern Chinese can be shared upon reasonable request by contacting the corresponding authors.
