# Supplementary material for: Exploratory Analysis of the Association Between Plasma Ceramide Alterations and Cognitive Dysfunction in Parkinson's Disease
Source: CNS Neurosci Ther. 2024 Oct 20;30(10):e70082. doi: 10.1111/cns.70082 (PMC11491299; doi:10.1111/cns.70082)

**Exploratory analysis of the association between plasma ceramide alterations and cognitive dysfunction in Parkinson's disease**

Supplementary information

Xu Liu^1,2,3#^, Xuanjing Liu^3#^, Yuning Liu^1,3^, Bo Yang^3^, Yangdanyu Li^1,3^, Fujia Li^1,3^, Kun Qian^1,3^, Xuesong Liu^3^, Lishun Xiao^4^, Guiyun Cui^1,3*^, Chuanying Xu^1,3*^

^1^Department of Neurology, The Affiliated Hospital of Xuzhou Medical University, 99 West Huaihai Road, Xuzhou, Jiangsu Province, 221000, China

^2^Department of Neurology, The Second Affiliated Hospital of Xuzhou Medical University, 32 Meijian Road, Xuzhou, Jiangsu Province, 221000, China.

^3^Department of Neurology, The First Clinical College, Xuzhou Medical University, 209 Tongshan Road, Xuzhou, Jiangsu Province, 221000, China

^4^Department of Biostatistics, School of Public Health, Xuzhou Medical University, 209 Tongshan Road, Xuzhou, Jiangsu Province, 221000, China

**^#^**These authors contributed equally:Xu Liu, Xuanjing Liu

***Corresponding Authors:** Chuanying Xu, Guiyun Cui

Email: xucy2022@yeah.net (C. Xu), teachercuigy@126.com (G. Cui)

Table S1 Demographic and clinical characteristics of all baseline subjects in the PPMI cohort.

| Variables | HC (N=125) | PD (N=304) | *P*-value |
| --- | --- | --- | --- |
| Age(years) | 62.61(13.71) | 62.28(14.17) | 0.914 |
| Male sex, n(%) | 86(68.80%) | 196(64.47%) | 0.434 |
| BMI, kg/m^2^ | 27.20(60.06) | 26.68(5.63) | 0.266 |
| Education, years | 16(3.5) | 16(4) | 0.104 |
| MoCA score | 28(2) | 27(3) | **<0.001** |
| Disease duration, years | NA | 0.36(0.46) | — |
| LEDD(mg) | NA | 0 | — |
| MDS-UPDRS III score | NA | 20(11.75) | — |
| H-Y stage | NA | 2(1) | — |
| Cer 16:0, umol/L | 0.09(0.03) | 0.09(0.03) | 0.320 |
| Cer 18:0, umol/L | 0.03(0.02) | 0.03(0.01) | 0.110 |
| Cer 24:0, umol/L | 1.34(0.48) | 1.27(0.48) | 0.091 |
| Cer 24:1, umol/L | 0.66(0.24) | 0.70(0.27) | 0.076 |
| Cer 16:0/Cer 24:0 | 0.07(0.02) | 0.07(0.02) | **0.003** |
| Cer 18:0/Cer 24:0 | 0.02(0.01) | 0.03(0.01) | **0.004** |
| Cer 24:1/Cer 24:0 | 0.50(0.16) | 0.57(0.18) | **<0.001** |

Table S2 Demographic and clinical characteristics of 125 PD patients based on MoCA scores over the 3rd year of follow up in the PPMI cohort.

| Variables | MoCA score ≥ 25 (N=96) | MoCA score＜25 (N=29) |
| --- | --- | --- |
| Age(years) | 61.15(12.30) | 65.96(9.56)^a**^ |
| Male sex, n(%) | 64(66.67%) | 19(65.52%) |
| BMI, kg/m^2^ | 26.77(5.24) | 26.15(6.03) |
| Education, years | 16.00(3.25) | 16(4) |
| MoCA score | 27(3) | 22.5(2) |
| Disease duration, years | 3.45(0.57) | 3.34(0.56) |
| LEDD(mg) | 400.0(453.4) | 440.0(415.6) |
| MDS-UPDRS III score | 28.0(16.5) | 32.5(18.0) |
| H-Y stage | 2(0) | 2(1) |
| Cer 16:0, umol/L | 0.09(0.03) | 0.09(0.04) |
| Cer 18:0, umol/L | 0.03(0.02) | 0.03(0.02) |
| Cer 24:0, umol/L | 1.29(0.52) | 1.40(0.66) |
| Cer 24:1, umol/L | 0.66(0.28) | 0.79(0.23) |
| Cer 16:0/Cer 24:0 | 0.07(0.02) | 0.08(0.04) |
| Cer 18:0/Cer 24:0 | 0.02(0.01) | 0.02(0.01)^a*^ |
| Cer 24:1/Cer 24:0 | 0.54(0.22) | 0.57(0.17)^a*^ |

^a^MoCA scores < 25 vs MoCA score ≥ 25; ^*^ *P* < 0.05, ^**^ *P* < 0.01.

Note: In the PPMI cohort, there are only laboratory plasma ceramide data available for 3-years follow-up. After removing the missing values to ensure that plasma ceramide data are available for each follow-up time point, only 125 PD patients remain. Additionally, since the PPMI cohort consists entirely of de novo PD patients, the proportion of patients with cognitive impairment at baseline is very low, as an alternative, we hence chose to compare the levels of plasma ceramides based on MoCA scores at the 3rd-year follow-up time point.

Table S3 COX model to identify factors for PD-CD in 125 PD patients in the PPMI cohort during 1-3 years follow up.

|  | **Univariable analysis** | |
| --- | --- | --- |
| Variables | *B* | *P*-value |
| Age, years | 0.074 | **<0.001** |
| Sex | 0.066 | 0.817 |
| BMI, kg/m^2^ | 0.035 | 0.131 |
| LEDD(mg) | 0.001 | **0.018** |
| Cer 16:0, umol/L | 3.178 | 0.510 |
| Cer 18:0, umol/L | 12.62 | 0.219 |
| Cer 24:0, umol/L | -0.66 | **0.070** |
| Cer 24:1, umol/L | -0.381 | 0.578 |
| Cer 16:0/Cer 24:0 | 15.804 | **0.008** |
| Cer 18:0/Cer 24:0 | 28.132 | **0.012** |
| Cer 24:1/Cer 24:0 | 1.054 | 0.204 |

Fig.S1 Associations between proportion of normal cognition and plasma ceramides grouping by baseline median concentrations in Kaplan - Meier method in the PPMI cohort.


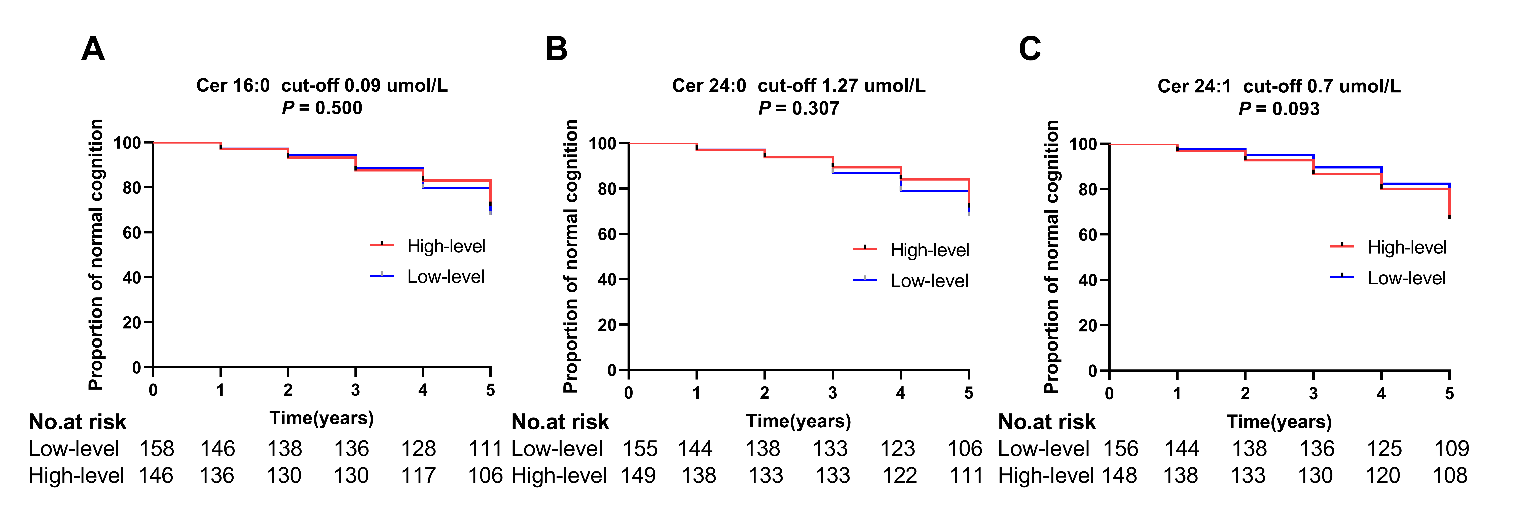

Supplement: Supplementary file 1 — Appendix S1. [file CNS-30-e70082-s001.docx]
